# Transport and Biotransformation of Gliclazide and the Effect of Deoxycholic Acid in a Probiotic Bacteria Model

**DOI:** 10.3389/fphar.2019.01083

**Published:** 2019-09-24

**Authors:** Maja Ðanić, Bojan Stanimirov, Nebojša Pavlović, Saša Vukmirović, Jelena Lazić, Hani Al-Salami, Momir Mikov

**Affiliations:** ^1^Department of Pharmacology, Toxicology and Clinical Pharmacology, Faculty of Medicine, University of Novi Sad, Novi Sad, Serbia; ^2^Department of Biochemistry, Faculty of Medicine, University of Novi Sad, Novi Sad, Serbia; ^3^Department of Pharmacy, Faculty of Medicine, University of Novi Sad, Novi Sad, Serbia; ^4^Biotechnology and Drug Development Research Laboratory, School of Pharmacy and Biomedical Sciences, Curtin Health Innovation Research Institute, Curtin University, Perth, WA, Australia

**Keywords:** gliclazide, gut microflora, bile acids, biotransformation, transport

## Abstract

**Introduction:** Inter-individual differences in gut microflora composition may affect drug metabolism and overall therapeutic response. Gliclazide is a drug characterized by large inter-individual differences in therapeutic response; however, the causes of these differences are not fully explained and may be the outcome of microbial biotransformation. Recently, great attention has been paid to studies on bile acid (BA) interactions with gut microflora and the role of BAs in the modification of drug transport through biological membranes.

**The Aim:** Considering the assumption of gliclazide–probiotic–BAs interactions, the aim of the study was to investigate the transport and biotransformation of gliclazide in probiotic bacteria, as well as the effects of deoxycholic acid (DCA) on gliclazide transport into bacterial cells.

**Materials and Methods:** Probiotics were incubated with gliclazide with or without DCA for 24 h at 37°C. The intracellular and extracellular concentrations of gliclazide were determined at seven time points by high-performance liquid chromatography. Gliclazide biotransformation by the enzymatic activity of probiotic bacteria was examined using appropriate software packages.

**Results:** During the 24 h incubation with probiotic bacteria, significantly lower extracellular concentrations of gliclazide were observed at all time points compared to controls, while in the group with DCA, the decrease in concentration was noticed only at 24 h. The total concentration of gliclazide throughout the whole period was significantly lower compared to control. Proposed pathways of gliclazide biotransformation by probiotic bacteria involve reactions of hydrolysis and hydroxylation.

**Conclusion:** Based on the results obtained, it can be concluded that there are interactions of gliclazide–probiotics–DCA, at both the level of active and passive transport into the cells, and at the level of drug biotransformation by enzymatic activity of probiotic bacteria. The effect of these interactions on the final therapeutic response of gliclazide should be further studied and confirmed in *in vivo* conditions.

**Figure:**
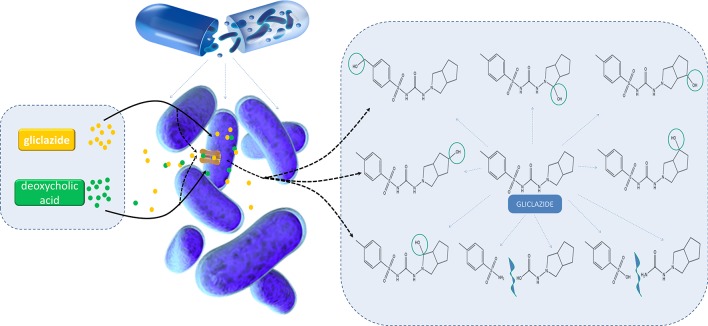
Graphical Abstract

## Introduction

Gut microflora is a complex ecosystem composed of various microorganisms, mostly bacteria, residing in or passing through the gastrointestinal tract (GIT). The composition of the intestinal microflora is unique and specific for each individual, and represents its so-called bacterial *fingerprint* (Stojančević et al., 2014). Although it is relatively stable throughout life, a wide range of factors such as changes in lifestyle and nutrition, stress, exposure to some drugs, and toxins and various diseases may lead to temporary or permanent disruption of the composition causing a state of dysbiosis. In this case, there are attempts to modify its structure and activity. One approach is the use of probiotics, in particular, bacteria that are normally present in GIT (Cresci and Bawden, 2015). Probiotics are living microorganisms that perform a health positive effect in the host when administered in an adequate dose (George Kerry et al., 2018). The most common probiotics include lactic acid bacteria and bifidobacteria, although other bacteria and certain yeasts are also used (Pandey et al., 2015).

Important roles of intestinal bacteria include the metabolism and absorption of nutrients, the metabolism of many endogenous substances, such as bile acids (BAs), bilirubin, cholesterol, steroid hormones, fatty acids, synthesis of vitamin K and B, modulation of the immune system, and protection against colonization by pathogenic bacteria (Shreiner et al., 2015; Thursby and Juge, 2017; Rowland et al., 2018). Recently, the role of intestinal microflora and probiotic bacteria in drug metabolism and drug response has attracted a great deal of attention among the scientific community (Klaassen and Cui, 2015; Wilson and Nicholson, 2017; Choi et al., 2018). As a result of the high metabolic capacity of intestinal microflora, physiologically active, inactive and even toxic metabolites of drugs may be formed (Stojančević et al., 2014). Therefore, understanding gut microflora-mediated drug metabolism is critical to interpreting changes in drug pharmacokinetics (Zhang et al., 2018). In addition to biotransformation *via* bacterial enzymes, drug interactions with intestinal bacteria are also possible at the transport level, due to the presence of a large number of so-called *multidrug* transporters on the membranes of intestinal bacteria (Ðanić et al., 2016a). Hence, the involvement of bacterial transporters may be one potential mechanism by which gut microflora affect drug pharmacokinetics (Choi et al., 2018).

Drugs which have low solubility and/or permeability, and reach the lower parts of the GIT where bacteria are the most abundant, are particularly good candidates for interactions with gut microflora. These interactions may define both the disposition profile and the pharmacologic activity of the drug (Sousa et al., 2008; Stojančević et al., 2014).

One such candidate is gliclazide, which belongs to the second generation of sulphonylurea derivates, drugs used in the treatment of diabetes mellitus type 2. Gliclazide achieves effects by selective binding to sulfonylurea receptors (SUR-1) on the surface of the pancreatic β-cells, which in turn leads to exocytosis and release of insulin from vesicles (Singh and Singh, 2016). In addition to the well-known role in type 2 diabetes, Mikov et al. (2018) highlighted the potential role of gliclazide in type 1 diabetes and pointed to the relationship between diabetes, gut microflora disturbance and BA secretion. In terms of chemical structure, gliclazide is a weak acid and a small lipophilic molecule that contains three chemical groups: an aromatic core, a sulfonylurea group, and an azabicyclic ring (Biswal et al., 2009). According to the biopharmaceutical classification system (BCS), gliclazide belongs to the second class of drugs characterized by low water solubility and high permeability. For this group of drugs, dissolution rate is a limiting factor for bioavailability (Biswal et al., 2009). Gliclazide is metabolized in the liver producing 7 metabolites *via* hydroxylation, N-oxidation and oxidation reactions (Oida et al., 1985). A number of reports have drawn attention to inter-individual variations in the bioavailability of gliclazide, which are partly explained by its poor aqueous solubility and unsatisfactory dissolution rate (Kobayashi et al., 1984; Davis et al., 2000; Holstein et al., 2011). In addition, one of the reasons for this variability might be its metabolism by intestinal microflora and probiotic bacteria, which will be the topic of this research, since, according to our knowledge, there are no data addressing this issue.

In recent years, a large number of studies have demonstrated that BAs may considerably affect drug transport through interfaces (Pahomi et al., 2012) and particularly through biological membranes that can greatly influence the efficacy of certain drugs, far surpassing the mechanical role of BAs as intestinal detergents (Stojančević et al., 2013, Pavlović et al., 2018). The physiological presence of BAs in the GIT, their structural modification by intestinal bacteria, and the role of BAs in drug formulation and delivery together (Stojančević et al., 2013; Pavlović et al., 2018) indicate that BA–probiotics–drug interactions may be expected. In addition, it has been claimed that the BAs themselves exert a hypoglycemic effect, which opens the possibility of using BAs as agents in the treatment of metabolic diseases, particularly diabetes mellitus (Ðanić et al., 2018).

In order to study the interactions of intestinal bacteria, BAs and gliclazide, and to predict the effect of these interactions on the therapeutic response of gliclazide, the aim of our study was to examine gliclazide transport into probiotic bacteria in *in vitro* conditions, the influence of deoxycholic acid (DCA) on the gliclazide transport into probiotic bacteria and to perform the *in silico* analysis of biotransformation of gliclazide by probiotic bacteria.

## Material and Methods

### Chemicals and Reagents

The probiotic used in this study was the commercial preparation PROBIOTIC^®^ (Hemofarm AD, Serbia). The capsules are declared to contain 5 × 10^9^ lyophilized cells of *Lactobacillus acidophilus* Rosell-52, *Lactobacillus rhamnosus* Rosell-11 and *Bifidobacterium longum* Rosell-175 strains. Bacterial strains have been identified and characterized by Pasteur Institute, France. To evaluate the accuracy of label claims, the number of viable bacteria of the probiotic product was pretested using traditional methods of cultivation confirming that the product met its label claim. Gliclazide was obtained from Hemofarm AD, Serbia and DCA from Sigma Chemicals Co, St Louis, MO, USA. Water, acetonitrile and dimethyl sulfoxide (DMSO) were of HPLC grade and obtained from J.T. Baker (Phillipsburg, NJ, USA). Phosphate-buffered saline (PBS) (0.01M, pH 7.4) was prepared by dissolving 8.0 g NaCl, 0.2 g KCl, 0.24 g KH_2_PO_4_, and 1.44 g Na_2_HPO_4_ in 1 L redistilled water and pH was adjusted to the 7.4 with HCl.

### Preparation of Working and Stock Standard Solutions

The stock solution of gliclazide (20 mg/mL) was prepared by dissolving the appropriate amount of gliclazide in DMSO. Standard gliclazide solutions for the calibration curve were prepared by diluting the stock gliclazide solution with the mobile phase to the final concentrations in the range of 0.2–100 μg/mL. The dependence of the peak area on the concentration was analyzed. The correlation coefficient of the calibration curve obtained as the dependence of the peak area on the concentration of gliclazide was R^2^ = 0.9994. The calibration curve equation was y = 0.9588x + 0.2158. A stock solution DCA at a concentration of 25 mM was made by dissolving the appropriate amount of DCA in DMSO.

### Protocol and Sample Preparation

The content of a half of the probiotic capsule was mixed and shaken with 5 mL of gliclazide solution in PBS buffer (200 μg/mL) in a test tube with a screw cap making suspension of probiotic bacteria (5 × 10^8^/mL). Experiments were performed with a submicellar concentration of DCA (0.25 mM) (Natalini et al., 2007). Experimental groups were labeled with GP and GPD (with DCA), respectively. Control groups (G, GD) were prepared in the same way but without probiotic bacteria in order to compare probiotic effect on gliclazide with the spontaneous degradation of gliclazide during the time.

The tubes were incubated in the dark at anaerobic conditions at 37°C for 24 h, gently shaking the tubes occasionally. The gliclazide concentrations in the extracellular, intracellular and total medium were determined at seven time points (0min, 30min, 1, 2, 4, 6, and 24 h).

For the analysis of the extracellular content, 100-μL aliquots were taken after gently shaking the tubes to uniformly distribute the contents, which were then centrifuged for 5 min at 15,000 rpm to precipitate bacteria. After that, in order to provide precipitation of the proteins, 50 μL of clear supernatant was diluted 5-fold with acetonitrile and centrifuged for 10 min at +4 ºC and at 15,000 rpm. The supernatant was transferred in a sample vial of the autosampler and 100 μL was directly injected in HPLC system.

For the analysis of intracellular content, precipitated bacteria that remained after the first step of centrifugation were used. Firstly, the remaining supernatant was carefully poured off. After that, bacterial cells were washed three times very gently with PBS and resuspended in 100 μL of deionized water followed by ultrasonic disruption. Sonication was performed for three 2-min intervals with 3-min rest intervals between in an ice bath (Mehmeti et al., 2013). After precipitation of bacterial cell debris, 50 μL of clear supernatant was diluted 5-fold with acetonitrile and centrifuged for 10 min at +4 ºC and at 15,000 rpm. The obtained supernatant was used as the intracellular fraction of SV that was analyzed by HPLC. During the analysis, 5-fold dilution with acetonitrile was considered. All experiments were performed in triplicate (using three batches of probiotic capsules). Total concentration was calculated as the sum of intracellular and extracellular concentration.

### HPLC Analysis

The analysis was performed by a high-performance liquid chromatography (HPLC; Dionex) with a diode array detector (DAD) according to the previously published method (Lalic-Popovic et al., 2013). The analysis was performed on a reverse-phase column Zorbax Eclipse Plus-C18 (100 *mm* × 2.1 mm, 5 μm, Agilent Technologies, USA), with precolumn Zorbax extend C18 (12.5 mm × 2.1 mm, 5 μm, Agilent Technologies, USA). During the analysis, the column temperature was kept constant (25°C) and the injection volume was 20 μL. The elution was performed by an isocratic program. The mobile phase consisted of acetonitrile and water (49:51% v/v) and the pH was adjusted with acetic acid to 2.7. The isocratic flow rate of mobile phase was maintained at 0.4 mL/min. The total duration of the analysis was 8 min. The eluate was monitored using a UV/DAD detector at a 229 nm wavelength.

### Bile Acid–Gliclazide Complexation — Structural Modeling and Geometry Optimization

Initial 3D structures of gliclazide and DCA were constructed using Chem3D Ultra v. 16.0.1.4 program package. Initial 3D structure of their complex was prepared in the same way. Molecular geometries were optimized using MM2 force field calculations, as implemented in Chem3D Ultra software. After optimization, total energies of analyzed compounds and their complexes were calculated.

### *In Silico* Methods for Predicting the Pathways of Gliclazide Biotransformation by the Activity of Bacterial Enzymes

Potential pathways of gliclazide biotransformation were predicted by *in silico* methods using appropriate software packages. Online tool MetaPrint2D was used to predict potential sites of metabolism in the chemical structure of gliclazide by uploading the SMILES string of gliclazide. Apart from identification of sites of metabolism, MetaPrint2D is able to predict types! of transformation and the likely metabolites formed. Using this tool, the atoms in the gliclazide molecule that are most susceptible to changes, as well as the reactions that can be performed on them, were marked. At the same time, to each prediction it generates the Normalized Occurrence Ratio (NOR) value is assigned, which represents the probability of enzymatic reaction on certain atom (Carlsson et al., 2010; MetaPrint2D).

Additionally, in order to further investigate the effect of enzymatic activity of examined probiotic bacteria on the gliclazide biotransformation pathways, the EAWAG-BBD Pathway Prediction System was used (EAWAG-BBD Pathway Prediction System, 2010). The predictions are based on the reactions and rules available in the database, as well as based on existing literature data.

### Statistics

Statistical analysis of experimental results was performed by the statistical program IBM SPSS Statistics, ver. 21. Analysis concerned triplicate results. All data were expressed as mean ± standard deviation (SD). A value of p < 0.05 was considered to be statistically significant. The statistical significance of the difference between the average values of the parameters was tested by a One-way ANOVA with post-hoc Tukey HSD for simultaneous comparison of multiple samples, and a One-way ANOVA test of repeated measures with the Sidak test for comparing different time points within the same group.

## Results

### Gliclazide Penetration Into Probiotic Bacteria and the Effect of Deoxycholic Acid on the Penetration of Gliclazide Into Probiotic Bacteria

**Figure 1:**
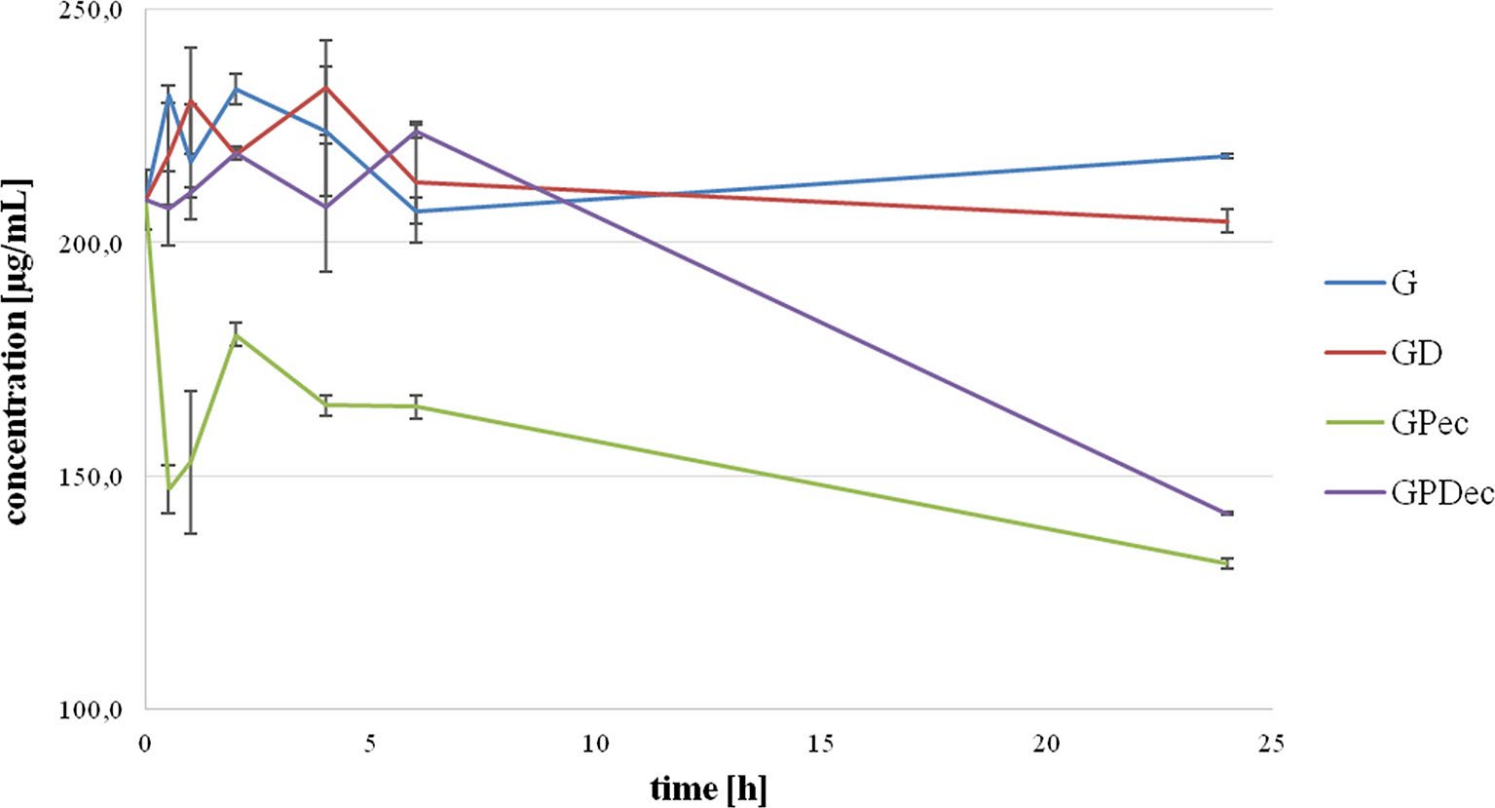
Extracellular gliclazide concentration during incubation with probiotics, without (GPec) and in the presence of deoxycholic acid (GPDec), compared with their control groups (G) and (GD), respectively.

**Figure 1** shows the extracellular gliclazide concentration during a 24-hour incubation with probiotics without DCA (GPec) and in the presence of DCA (GPDec) compared with their control groups, G and GD (without probiotic bacteria, respectively).

It can be observed that gliclazide concentrations in control groups were relatively stable over the whole observed period, and there were no statistically significant differences between the control groups, G and GD. On the other hand, during the incubation with probiotic bacteria, a statistically significant decrease in extracellular gliclazide concentration was noted, which was mostly expressed during the first 30 min (from 209.16 ± 6.26 μg/mL at 0 min to 147.11 ± 5.08 μg/mL at 30 min, p < 0.05). After this rapid drop, the concentration was maintained at a relatively constant level over time, with another significant drop after 6 h, reaching the concentration 131.21 ± 1.17 μg/mL at 24 h. Concentrations of gliclazide incubated with bacteria and DCA were not markedly different from controls during the first 6 h, while at 24 h a statistically significant difference was registered (204.60 ± 2.48 µg/mL in GD and 141.95 ± 0.42 µg/mL in GPDec, p < 0.05).

**Figure 2:**
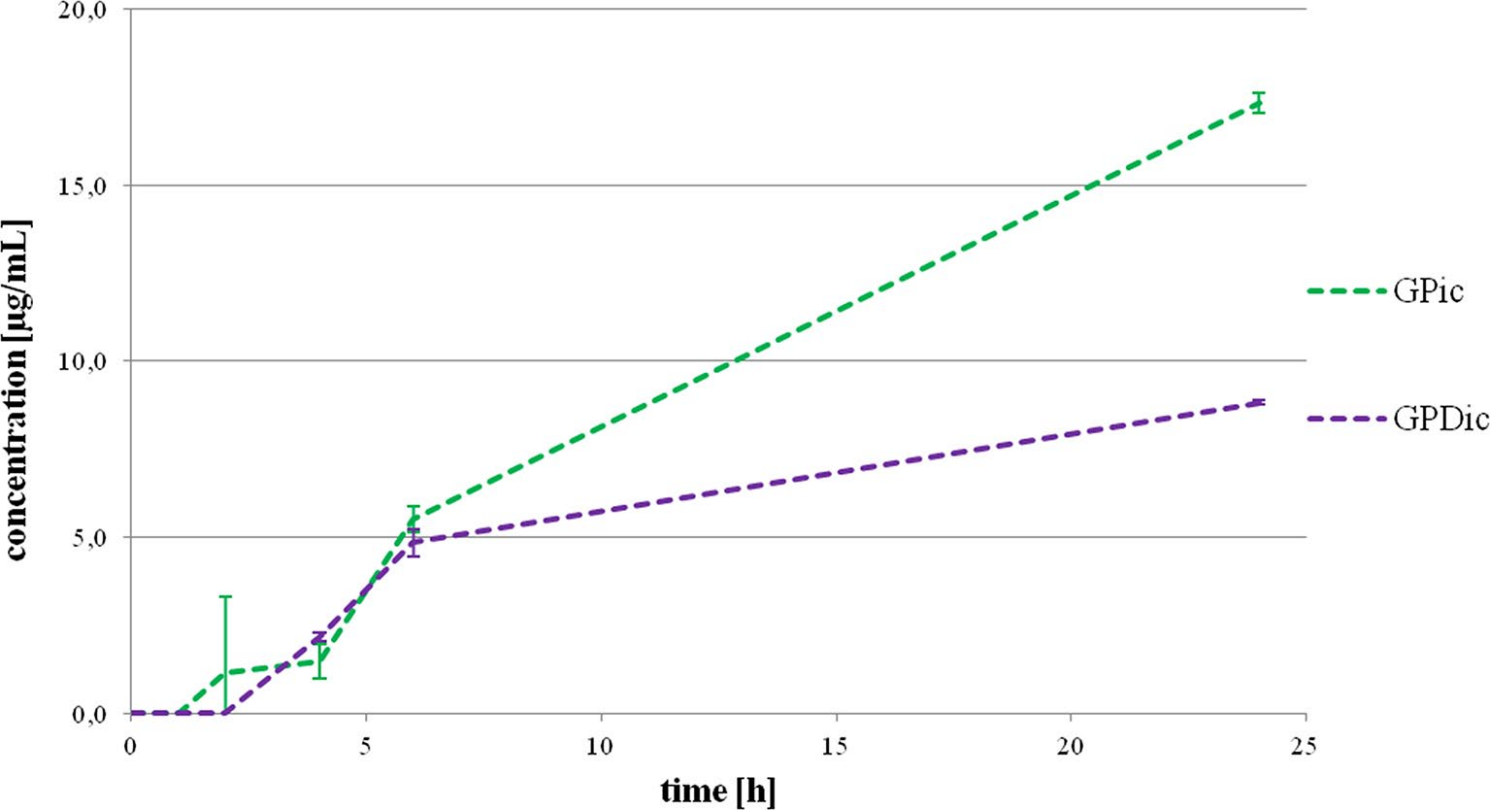
Intracellular gliclazide concentration during a 24-h incubation with probiotics without the presence (GPic) and in the presence of deoxycholic acid (GPDic).

**Figure 2** illustrates intracellular gliclazide concentrations during a 24-h incubation with probiotics, without (GPic) and in the presence of DCA (GPDic). In the group with probiotic bacteria but without DCA, the presence of gliclazide in the intracellular content is evident from the second hour. The intracellular gliclazide concentration mildly rises until 6 h. The highest increase is recorded from 6 to 24 h (from 5.51 ± 0.36 μg/mL to 17.33 ± 0.29 μg/mL, respectively, p < 0.05). In the group with DCA, intracellular concentrations of gliclazide were lower compared to the group without DCA, reaching the value of 8.82 ± 0.06 μg/mL at 24 h.

**Figure 3:**
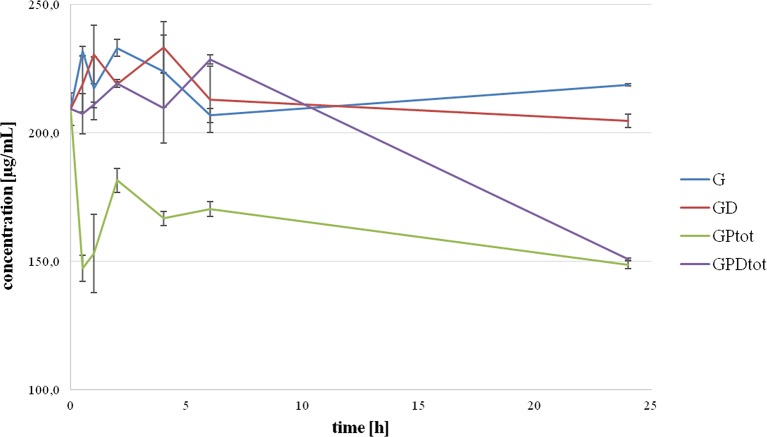
Total concentration of gliclazide as a sum of intracellular and extracellular concentration during incubation with probiotics without the presence (GPtot) and in the presence of deoxycholic acid (GPDtot) compared with their control groups (G) and (GD), respectively.

The total concentrations of gliclazide over the 24-hour incubation period with probiotics, without (GPtot) and with DCA (GPDtot), obtained as the theoretical sum of extracellular and intracellular concentrations are shown in **Figure 3**. It can be observed that total concentrations of gliclazide in the group with probiotics without DCA (GPtot) were significantly lower compared to the control in all time points. The total gliclazide concentration at 24 h was approximately 30% lower compared to the initial concentration. On the other hand, in the group with probiotics and DCA (GPDtot), there were no large differences compared to the control group over the first 6 h of incubation; however, at 24 h of incubation, the total gliclazide content fell rapidly and had values that were not significantly different from concentrations in the group without DCA (GPtot). During the whole incubation period, there were no statistically significant differences between the total concentration in the control groups, G and GD. Chromatograms for all tested groups are provided as **Supplementary Data**.

### Bile Acid–Gliclazide Complexation

**Figure 4:**
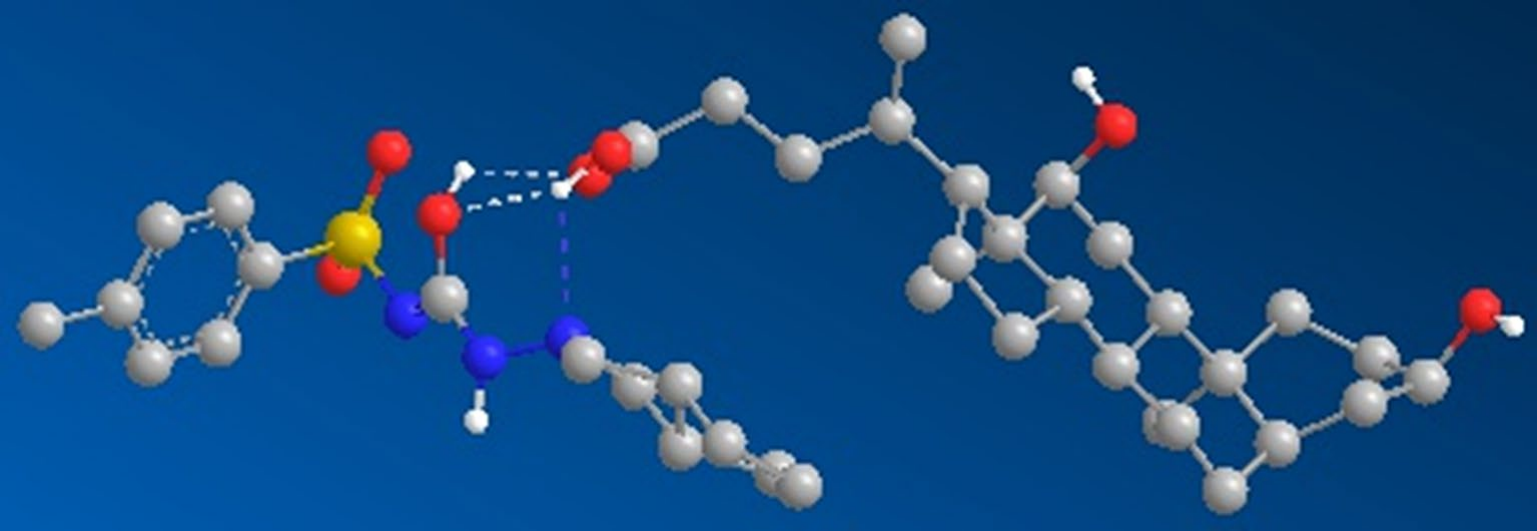
Geometrically optimized three-dimensional structure of the gliclazide-DCA complex.

The interactions of gliclazide and DCA were investigated by molecular mechanics calculations (MM2) using the geometrically optimized 3D structures (**Table 1**). The minimized total energies of gliclazide and DCA were 215.53 kcal/mol and 55.21 kcal/mol, respectively. The total energy of the gliclazide/DCA complex (250.35 kcal/mol) was lower than the sum of the potential energies of the two single components optimized by molecular mechanics calculations, indicating that the formation of the complex induced a stabilization of the system. For the formation of the gliclazide/DCA complex the main contribution was supplied by electrostatic attraction forces and hydrogen bonds (**Figure 4**).

**Table 1:** The minimized total energies of gliclazide, DCA and their complex.

E_G_ (kcal/mol)	E_DCA_ (kcal/mol)	E_G_ + E_DCA_ (kcal/mol)	E_COMPLEX_ (kcal/mol)	ΔE (kcal/mol)
215.5275	55.2088	270.7363	250.3490	−20.3873

### Results of the *in Silico* Analysis of Gliclazide Biotransformation Pathways

**Figure 5:**
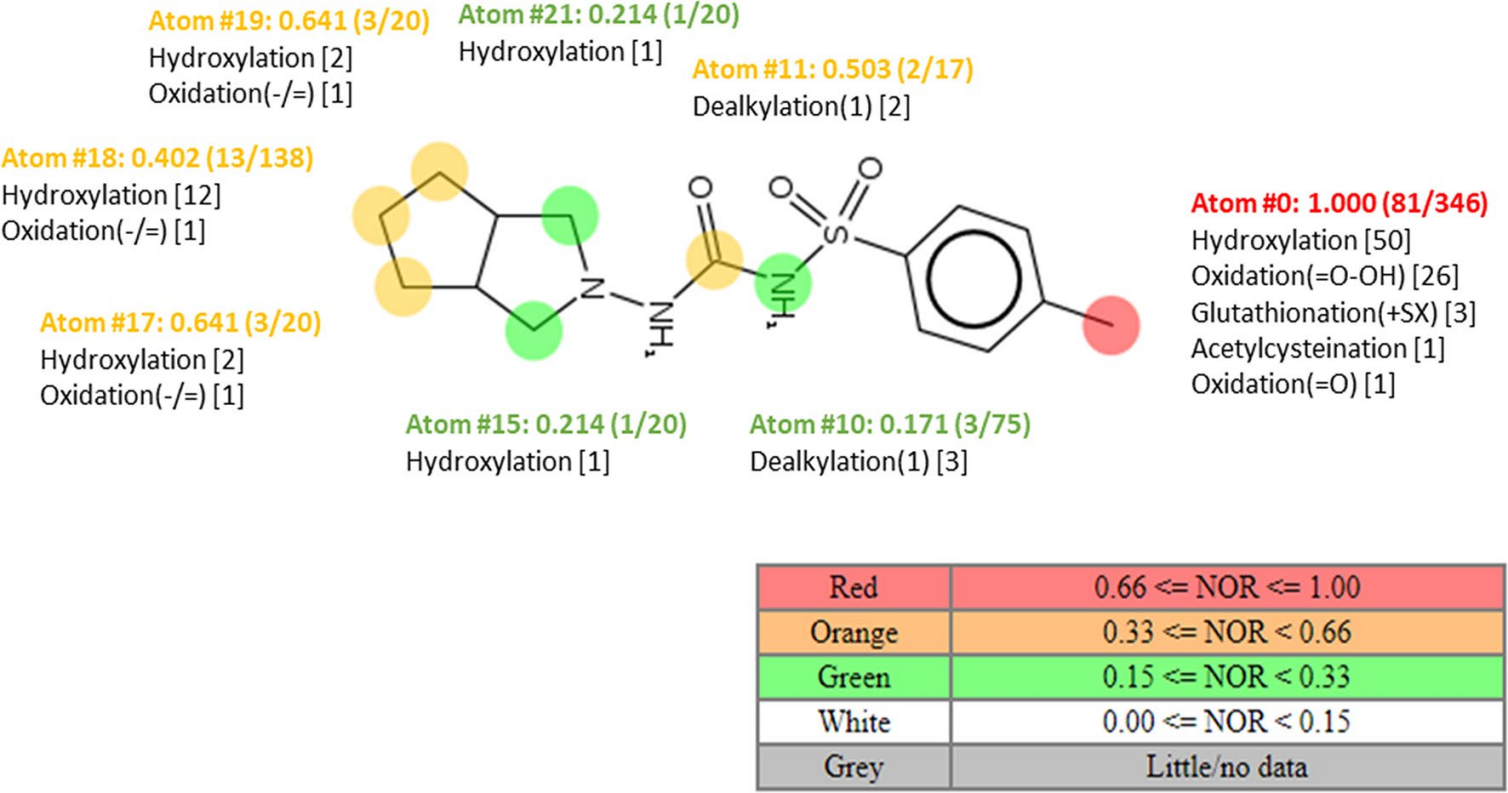
Plot of MetaPrint2D predictions. The atoms in the gliclazide molecule, which are most likely to be metabolized, as well as the reactions that potentially can take place are marked. NOR indicates the normalized occurrence ratio; a high NOR indicates a more frequently reported site of metabolism in the metabolite database.

Information on the potential pathways of enzymatic biotransformation of gliclazide produced by probiotic bacteria was obtained by *in silico* analysis. Using MetaPrint2D program, the atoms in the gliclazide molecule that are most likely to be metabolized are marked, as well as reactions that can take place, and NOR values are given (**Figure 5**). The most likely reaction sites are those that have the highest NOR values (colored in red), followed by the group colored in yellow and then in green. Notably, the NOR value does not indicate the likelihood that the test compound will be metabolized, but rather the relative probability of metabolism at a particular site, assuming that the molecule is metabolized.

According to **Figure 5**, in the gliclazide molecule, the methyl group attached to the aromatic ring is most likely to be metabolized (colored in red). Suggested reactions are hydroxylation, oxidation, glutathionylation, and acetylcysteination.

**Figure 6:**
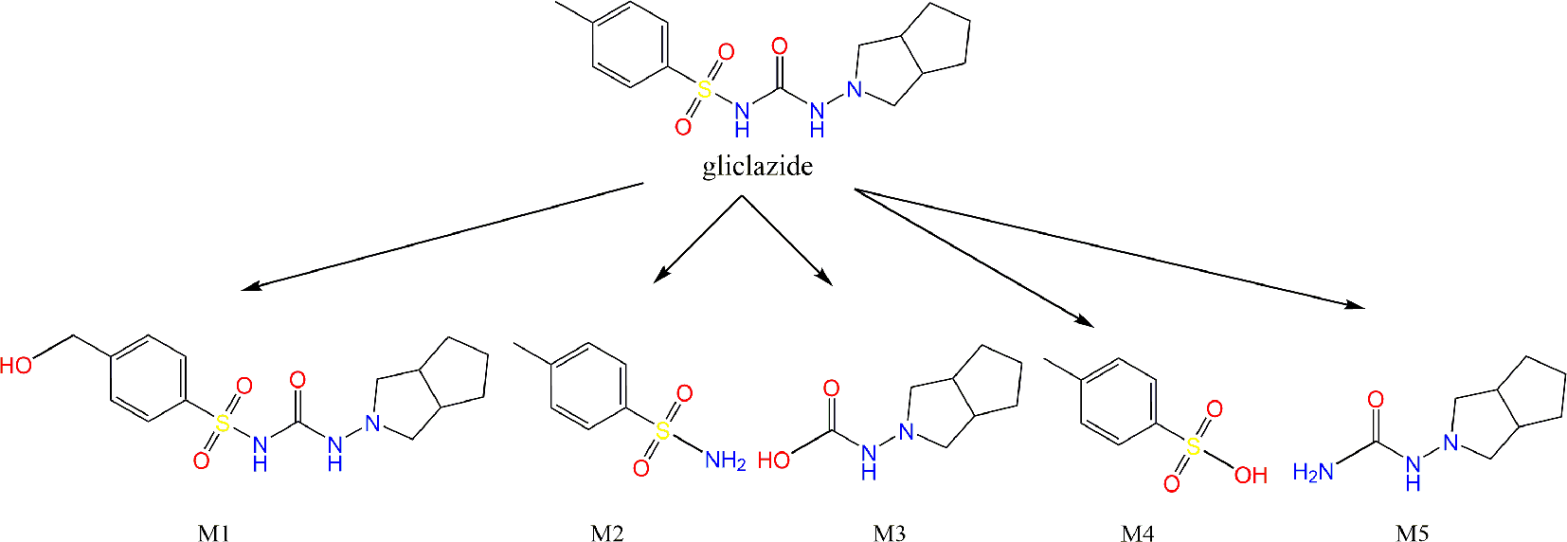
Results obtained by EAWAG-BBD pathway prediction system. The metabolites obtained by hydroxylation (M1), hydrolysis of C–N (M2 and M3) and S–N bonds (M4 and M5) are shown.

As a result of the *in silico* analysis by the EAWAG-BBD Pathway Prediction System program, possible reactions are hydroxylation, resulting in the formation of metabolite M1, and C–N and S–N hydrolysis, giving the metabolites M2 and M3, M4 and M5, respectively (**Figure 6**).

## Discussion

Due to inter-individual differences in the therapeutic response to gliclazide on the one hand (Holstein et al., 2011) and a great interest in gut microflora implications in drug metabolism and therapeutic effects on the other, the aim of this research was to examine the effect of intestinal flora and probiotics on the biotransformation of gliclazide *in vitro*. It has been already reported that administration of probiotics in rats affected gliclazide absorption and its systemic concentrations. In addition, probiotics also affected gliclazide transport across ileal tissue mounted in Ussing chambers (Al-Salami et al., 2008). Therefore, in order to explain these results, the aim of this study was to elucidate interactions of gliclazide and probiotic bacteria in more detail, to examine the transport of the drug into bacteria and its metabolism by bacterial enzymes. Due to the physiological presence of BAs in the GIT, their interactions with the intestinal microflora, their role in the modification of drug transport through biological membranes (Stojančević et al., 2013), and their hypoglycemic effect (Ðanić et al., 2018), the effect of a representative BA, DCA, on gliclazide transport into the probiotic bacteria was examined.

### Gliclazide Penetration Into Probiotic Bacteria

The results of the study showed that gliclazide concentrations in control groups without probiotic bacteria remained relatively stable over the incubation period, indicating that there is no spontaneous degradation of the drug over time, and also that DCA itself has no effect on the degradation of this drug. Analyzing the extracellular concentration of gliclazide in the experimental group with probiotic bacteria, a significant drop in concentration was observed over time. At the same time, measurable quantities of gliclazide were noticed in the intracellular content. From these results, it appears that gliclazide is transported from the extracellular space across the plasma membrane into bacterial cells over time. As a small lipophilic molecule, gliclazide passes through biological membranes by passive diffusion and partly by active transport (Mikov et al., 2018). *In vitro* and *in vivo* studies have shown that many oral antidiabetic drugs are substrates for uptake transporters (e.g., members of the SLC superfamily of solute carriers) and efflux proteins (e.g., members of the ABC transporter superfamily) expressed in the intestine, the liver and the kidney (Klatt et al., 2011). Due to the existence of homologous transporters in bacterial species (Ðanić et al., 2016a), it is important to highlight the possibility of active transport of gliclazide into bacterial cells, this being of great importance with regard to interactions with other substances and the consequential impact on pharmacokinetics of concomitantly taken drugs.

However, there is some kind of discrepancy related to extracellular and intracellular concentrations. Namely, the increase in intracellular concentration did not completely correspond to the decrease in extracellular concentration, implying that a fraction of the drug was metabolized (discussed further). In addition, the total concentration of gliclazide in the group with probiotic bacteria was lower compared to the control group, supporting this assumption.

### The Effect of DCA on the Gliclazide Penetration Into Probiotic Bacteria

In recent years, a number of studies have demonstrated the role of BAs in modifying drug transport through biological membranes. The main advantage of using BAs for that purpose is their ability to act as both drug solubilizing and permeation-modifying agents. In addition to micellar solubilization, there are many other types of interactions between bile acids and drug molecules which can influence drug transport across biological membranes, affecting both paracellular and transcellular transport (Stojančević et al., 2013; Pavlović et al., 2018).

In order to avoid possible membranolytic and bactericidal effects of DCA, submicellar concentrations were used. Watanabe et al. (2017) have shown that DCA caused no severe membrane damage or viability loss in *Bifidobacterium breve* Japan Collection of Microorganisms (JCM) 1192^T^ at similar concentrations to those used in this study (0.2 mM, membrane integrity 87.75%, viability 81.84%). These results suggest that the bactericidal activities determined against *B. breve* JCM 1192^T^ could be generalized to other bacterial species commonly represented in the human gut microbiota. Therefore, no bactericidal or membranolytic activity of DCA at concentrations used in our experiments were anticipated.

The results of our study showed that in the group with probiotics and DCA, a decrease in the extracellular gliclazide concentration was only evident after 24 h, with a complementary increase in intracellular content. Reduced transport of the drug into the cells in the presence of DCA can be discussed from the aspect of passive and active transport. One explanation may be competition for the same transport proteins by DCA, blocking drug transport into the cells. A recently published study has shown that the binding of the BAs to the so-called *multidrug* transporters in intestinal bacteria can lead to interactions with drugs, influencing their pharmacokinetics (Ðanić et al., 2016a). The transport of gliclazide into bacterial cells after 6 h can be explained by saturation of the transporters, after which the passive diffusion of gliclazide into the cells is likely to prevail. This explanation is supported by recently published results of docking studies confirming the interactions of BAs and drugs at transporter level in the *Lactobacillus* and *Bifidobacterium* species (Ðanić et al., 2016a). In addition to the modification of active drug transport, as mentioned above, BAs may affect the passive diffusion through the membranes as well (Pavlović et al., 2018). This can be explained by the formation of a large complex between gliclazide and DCA with hydrogen bonds stabilizing the complex, with two OH groups oriented toward the outer side of the complex. Hence, in addition to enhanced stability, the formed aggregate is expected to be more hydrophilic than the gliclazide molecule itself, with diminished affinity for the lipid membrane and impaired penetration into the cell by passive diffusion. Given that the formation of this complex is reversible, the free fraction of gliclazide was able to pass through the bacterial membrane, shifting the equilibrium towards the degradation of the complex. After 24 h, almost all the gliclazide was released from the complex with DCA into the free form able to penetrate into probiotic cells, where it was metabolized, resulting in similar concentrations to the probiotics group without DCA. However, in the group with DCA concentrations were still slightly higher extracellularly and slightly lower intracellularly suggesting that even after 24 h, not all gliclazide was released from the complex with DCA.

A similar conclusion was reached by Ðanić et al. (2016b), showing the increased water-solubility of simvastatin–DCA complex compared to simvastatin itself. Due to the fact that highly lipophilic properties of simvastatin are accompanied by low systemic bioavailability, BAs could be used to enhance the bioavailability of simvastatin and other drugs with similar properties. These findings are directly in line with the results of a study conducted by Kim et al. (2011) in which DCA has been shown to increase the water-solubility of lovastatin, which is also characterized by high lipophilicity, leading to an increase in its bioavailability in *in vivo* conditions. Since a major drawback in the therapeutic application and efficacy of gliclazide in oral dosage form is its very low water-solubility because of its hydrophobic nature (Özkan et al., 2000), it can be concluded that there is a possibility of increasing the bioavailability of gliclazide by forming hydrophilic aggregates with DCA. This could be useful for the development of new pharmaceutical formulations of gliclazide with BAs, having improved solubility and pharmacokinetic properties. In addition, it has been shown that BAs themselves exert a hypoglycemic effect, mostly through the activation of nuclear farnesoid X receptor (FXR) and the membrane TGR5 receptor signaling pathways, which opens the possibility of using BAs as agents in the treatment of metabolic diseases, primarily diabetes mellitus (Ðanić et al., 2018).

### Biotransformation of Gliclazide in Probiotic Bacteria — *In Silico* Analysis

In contrast to the liver, that is predominantly responsible for oxidative and conjugative reactions, modifications and/or metabolism by gut microflora and probiotic bacteria are mostly based on reduction and hydrolysis. In addition, gut bacteria express various enzymes involved in the reactions such as decarboxylation, dehydroxylation, dealkylation, acetylation, and deacetylation, denitration, mono-/di-oxygenation, N-demethylation, dehalogenation, deamination, opening of the thiazole ring, and the metabolism of glutathione conjugate xenobiotics secreted into the bile (Sousa et al., 2008; Wilson and Nicholson, 2017).

Considering that the total amount of gliclazide in the group with probiotic bacteria decreased significantly over time, we can conclude that gliclazide was partially metabolized by bacterial enzymes, indicating that not only physical interactions between gliclazide and DCA were present, but also biotransformation. In order to examine whether this decrease in gliclazide concentration was a consequence of degradation, we added control groups without probiotics: gliclazide alone and gliclazide + DCA, and no degradation was observed. Therefore, all concentration changes should be a consequence of microbial metabolism.

Since all metabolizing enzymes are located intracellularly, the previously discussed uptake of gliclazide from the extracellular space across the plasma membrane into the cell is a prerequisite for subsequent metabolism.

The biotransformation of gliclazide was lower in the group with DCA, where a higher total amount of gliclazide was noticed after incubation compared to the group without DCA, suggesting a protective role of DCA to hinder the access of bacterial enzymes to gliclazide.

Predictions from appropriate software suggest the most likely enzymatic activity towards gliclazide molecules catalyzed by probiotic bacteria include hydroxylation and hydrolysis reactions. The hydrolysis may take place at different sites in the gliclazide molecule: hydrolysis of C–N and S–N bonds, splitting the gliclazide molecule into two parts. These reactions may be catalyzed by hydrolases classified into EC 3.5 and EC 3.10 groups, which are commonly found in probiotic bacteria. One of the *first observations* of drug biotransformation *via* hydrolysis was methotrexate metabolism by the intestinal flora of normal mice (Zaharko et al., 1969). Hydrolytic enzymes produced by the gut microflora play a significant role in the activation of some orally administered prodrugs in the form of phosphate or sulphate esters which are used to improve poor biopharmaceutical properties of drugs, particularly solubility (Wilson and Nicholson, 2017).

Although examples of gut microbial oxidation/dehydrogenation are rare, a potential gut microbiota-mediated drug-drug interaction between lovastatin and antibiotics in rats (Yoo et al., 2014) and biotransformation of the dietary carcinogen 2-amino-3,6-dihydro-3H-imidazo,[4,5-f]quinoline to its 7-hydroxy metabolite (Bashir et al., 1987) were highlighted as examples in support of the hypothesis that the bacterial metabolism of gliclazide may be oxidative *via* hydroxylation as suggested by appropriate *in silico* methods. Hydroxylated metabolites, with additional hydroxyl groups, are expected to be more hydrophilic compared to the gliclazide molecule itself, which might be of crucial importance for better solubility and oral bioavailability.

The formation of proposed metabolites might be the explanation for the results obtained by Golocorbin-Kon et al. (2017) who showed that hypoglycaemic effects of gliclazide are not dependent on its high serum concentrations but rather gut metabolism activation. In order to confirm the proposed pathways of biotransformation of gliclazide, and to examine the effect of resulting metabolites, it is necessary to conduct additional *in vitro* and *in vivo* research.

## Conclusion

It can be concluded that there are interactions between gliclazide, BAs, and probiotics. The results of the *in vitro* experiment showed that during the 24-hour incubation of gliclazide with probiotic bacteria from the genus *Lactobacillus* and *Bifidobacterium*, some gliclazide is transported into probiotic bacteria. It has been shown that DCA affects the transport of the drug by competing for the same transporters, blocking the active transport on the one hand, and forming a hydrophilic complex and influencing passive diffusion on the other. Due to the complexity of the transport processes through the cell membrane and numerous factors that affect it, additional *in vivo* studies are needed to confirm the effects of these interactions.

In addition, the results of the study show that the total amount of gliclazide in the extracellular and intracellular content decreases over time compared to control, suggesting that the drug is partially metabolized by probiotic enzymes. According to the results of *in silico* analysis, the most likely reactions that take place in the gliclazide molecule are hydroxylation and hydrolysis. Further identification and confirmation of potential metabolites formed in this way, as well as the analysis of their potential pharmacological activity are of great importance, considering the possibility of changing the known metabolic pathway in the organism, and therefore the therapeutic response. Taking into account the fact that probiotic bacteria are a normal part of gut microflora and that each individual has a specific bacterial fingerprint, more attention should be paid to research that would further clarify the role of gut microflora in the metabolism of drugs and therapy individualization.

## Data Availability

Datasets generated for this study are included in the manuscript/**Supplementary Files**.

## Author Contributions

MÐ performed the literature search and drafted the manuscript. MÐ, BS and JL performed the experiments. MÐ, NP and SV contributed to designing the experiments, analyzing and interpreting the data. HA-S and MM provided critical revisions. All authors reviewed and approved the manuscript.

## Funding

This research was supported by HORIZON2020 MEDLEM project No. 690876, The Project for Scientific and Technological Development of Vojvodina No. 114-451-2072-/2016 and the project of Education, Science and Technology Development of the Republic of Serbia No. 41012.

## Conflict of Interest Statement

The authors declare that the research was conducted in the absence of any commercial or financial relationships that could be construed as a potential conflict of interest.

## Supplementary Material

Figure S1 Chromatograms of extracellular and intracellular gliclazide without (GPec24h and GPic24h, respectively) and with deoxycholic acid (GPDec24h and GPDic24h) after 24h incubation compared to controls (without probiotic bacteria) (G24h and GD24h, respectively).
