# Supplementary figures and images for: Transport and Biotransformation of Gliclazide and the Effect of Deoxycholic Acid in a Probiotic Bacteria Model
Source: Front Pharmacol. 2019 Sep 24;10:1083. doi: 10.3389/fphar.2019.01083 (PMC6771299; doi:10.3389/fphar.2019.01083)

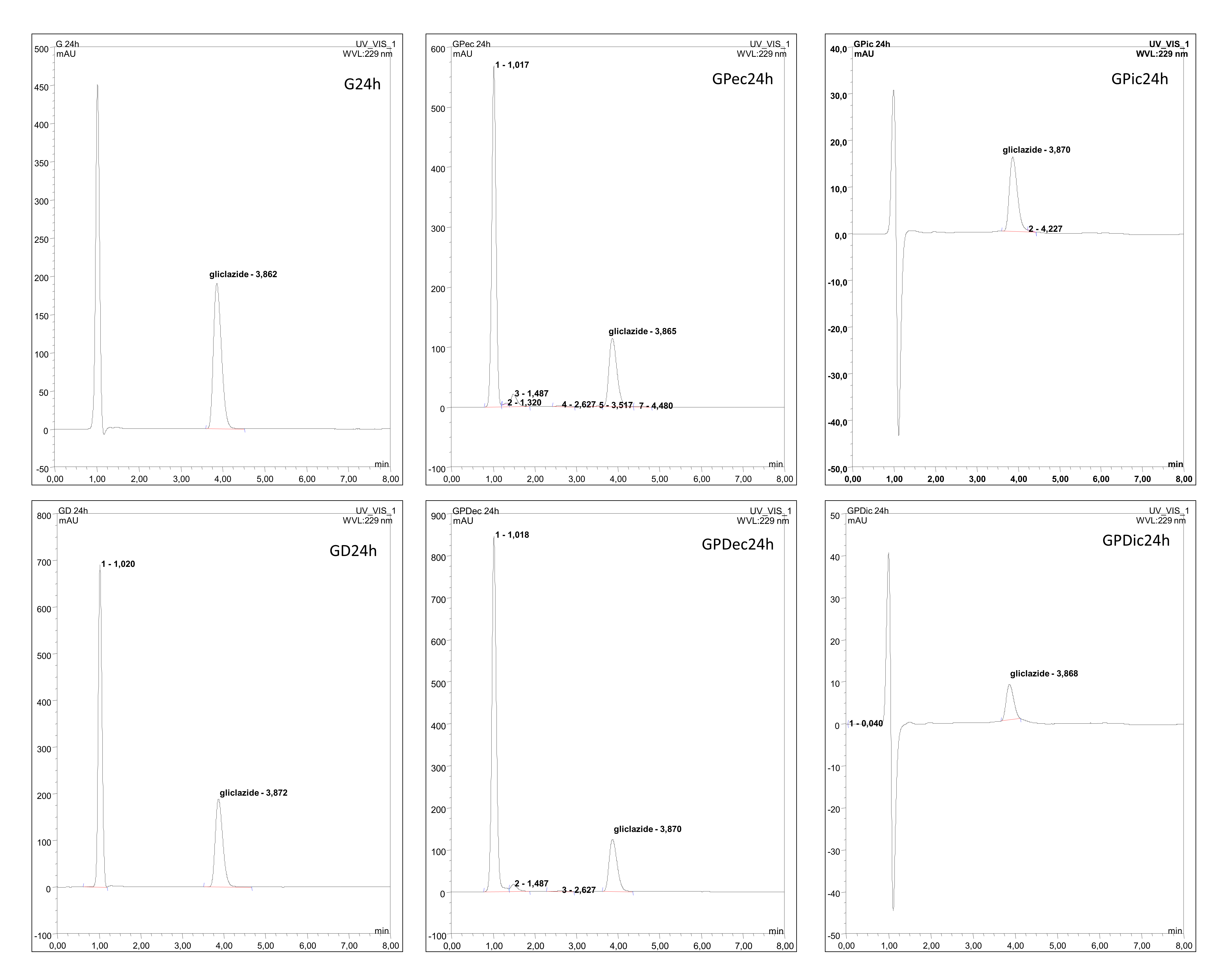

Supplement: Figure S1 — Chromatograms of extracellular and intracellular gliclazide without (GPec24h and GPic24h, respectively) and with deoxycholic acid (GPDec24h and GPDic24h) after 24h incubation compared to controls (without probiotic bacteria) (G24h and GD24h, respectively). [file Image_1.tif]
